# Effects of Environmental Factors on Severity and Mortality of COVID-19

**DOI:** 10.3389/fmed.2020.607786

**Published:** 2021-01-20

**Authors:** Domagoj Kifer, Dario Bugada, Judit Villar-Garcia, Ivan Gudelj, Cristina Menni, Carole Sudre, Frano Vučković, Ivo Ugrina, Luca F. Lorini, Margarita Posso, Silvia Bettinelli, Nicola Ughi, Alessandro Maloberti, Oscar Epis, Cristina Giannattasio, Claudio Rossetti, Livije Kalogjera, Jasminka Peršec, Luke Ollivere, Benjamin J. Ollivere, Huadong Yan, Ting Cai, Guruprasad P. Aithal, Claire J. Steves, Anu Kantele, Mikael Kajova, Olli Vapalahti, Antti Sajantila, Rafal Wojtowicz, Waldemar Wierzba, Zbigniew Krol, Artur Zaczynski, Katarina Zycinska, Marek Postula, Ivica Lukšić, Rok Čivljak, Alemka Markotić, Johannes Brachmann, Andreas Markl, Christian Mahnkopf, Benjamin Murray, Sebastien Ourselin, Ana M. Valdes, Juan P. Horcajada, Xavier Castells, Julio Pascual, Massimo Allegri, Dragan Primorac, Tim D. Spector, Clara Barrios, Gordan Lauc

**Affiliations:** ^1^Faculty of Pharmacy and Biochemistry, University of Zagreb, Zagreb, Croatia; ^2^Emergency and Intensive Care Department, Azienda Socio Sanitaria Territoriale Papa Giovanni XXIII Hospital, Bergamo, Italy; ^3^Hospital del Mar-Institut Hospital del Mar d'Investigacions Mèdiques, Barcelona, Spain; ^4^Genos Glycoscience Research Laboratory, Zagreb, Croatia; ^5^Department of Twin Research and Genetic Epidemiology, King's College London, London, United Kingdom; ^6^Faculty of Science, University of Split, Split, Croatia; ^7^Azienda Socio Sanitaria Territoriale Grande Ospedale Metropolitano Niguarda, Milan, Italy; ^8^School of Medicine and Surgery, Milano-Bicocca University, Milan, Italy; ^9^Department of Otolaryngology-Head and Neck Surgery, Zagreb School of Medicine, University Hospital Centre “Sestre milosrdnice”, Zagreb, Croatia; ^10^Clinical Department of Anesthesiology, Reanimatology and Intensive Care Medicine, University Hospital Dubrava Zagreb, Zagreb, Croatia; ^11^University of Zagreb School of Dental Medicine, Zagreb, Croatia; ^12^National Institute for Health Research Nottingham Biomedical Research Centre, Nottingham University Hospitals National Health Service Trust and the University of Nottingham, Nottingham, United Kingdom; ^13^Key Laboratory of Diagnosis and Treatment of Digestive System Tumors of Zhejiang Province, Department of Infectious Diseases, Hwamei Hospital, University of Chinese Academy of Sciences, Ningbo, China; ^14^Ningbo Institute of Life and Health Industry, University of Chinese Academy of Sciences, Ningbo, China; ^15^Inflammation Centre, Helsinki University Hospital, University of Helsinki, Helsinki, Finland; ^16^Department of Virology, Helsingin ja Uudenmaan Sairaanhoitopiiri Diagnostic Center, Helsinki University Hospital, University of Helsinki, Helsinki, Finland; ^17^Department of Veterinary Biosciences, University of Helsinki, Helsinki, Finland; ^18^Department of Forensic Medicine, University of Helsinki, Helsinki, Finland; ^19^Forensic Medicine Unit, Finnish Institute for Health and Welfare, Helsinki, Finland; ^20^Central Clinical Hospital of Ministry of the Interior and Administration, Warsaw, Poland; ^21^Medical University of Warsaw, Warsaw, Poland; ^22^Department of Experimental and Clinical Pharmacology, Center for Preclinical Research and Technology CEPT, Medical University of Warsaw, Warsaw, Poland; ^23^University of Zagreb School of Medicine, University Hospital Dubrava, Zagreb, Croatia; ^24^University Hospital for Infectious Diseases “Fran Mihaljević”, University of Zagreb School of Medicine, Zagreb, Croatia; ^25^University Hospital for Infectious Diseases “Fran Mihaljević”, Catholic University of Croatia, Zagreb, Croatia; ^26^Medical School, University of Rijeka, Rijeka, Croatia; ^27^REGIOMED Kliniken, Coburg, Germany; ^28^University of Split School of Medicine, Split, Croatia; ^29^School of Biomedical Engineering and Imaging Sciences, King's College London, London, United Kingdom; ^30^Pain Therapy Service Policlinico of Monza Hospital, Monza, Italy; ^31^St. Catharine Hospital, Zagreb, Croatia; ^32^Eberly College of Science, Penn State University, University Park, PA, United States; ^33^University of Osijek School of Medicine, Osijek, Croatia; ^34^Faculty of Dental Medicine and Health, University of Rijeka School of Medicine, University of Rijeka, Rijeka, Croatia

**Keywords:** COVID-19, seasonality, mortality, mucins, humidity

## Abstract

**Background:** Most respiratory viruses show pronounced seasonality, but for SARS-CoV-2, this still needs to be documented.

**Methods:** We examined the disease progression of COVID-19 in 6,914 patients admitted to hospitals in Europe and China. In addition, we evaluated progress of disease symptoms in 37,187 individuals reporting symptoms into the COVID Symptom Study application.

**Findings:** Meta-analysis of the mortality risk in seven European hospitals estimated odds ratios per 1-day increase in the admission date to be 0.981 (0.973–0.988, *p* < 0.001) and per increase in ambient temperature of 1°C to be 0.854 (0.773–0.944, *p* = 0.007). Statistically significant decreases of comparable magnitude in median hospital stay, probability of transfer to the intensive care unit, and need for mechanical ventilation were also observed in most, but not all hospitals. The analysis of individually reported symptoms of 37,187 individuals in the UK also showed the decrease in symptom duration and disease severity with time.

**Interpretation:** Severity of COVID-19 in Europe decreased significantly between March and May and the seasonality of COVID-19 is the most likely explanation.

## Background

Over a million COVID-19-related deaths have been reported until October 1, 2020, but a significant number of people (over 80% in some populations) infected with SARS-CoV-2 manage to contain infection in their upper respiratory tract and, despite being PCR positive for the viral RNA, do not develop any visible symptoms (1). So far, very little attention has been given to the effects of environmental conditions on the individual course of the diseases.

The first study of the environmental effects on the COVID-19 infection rate in 30 Chinese provinces found significant negative associations with temperature and relative humidity in Hubei province with the decrease of cases by 36–57% for every 1°C and 11–22% for every 1% increase in relative humidity; these associations were inconsistent in other provinces (2). Negative effects on COVID-19 transmission with warmer temperatures were also observed in Turkey (3), Mexico (4), Brazil (5), and United States (6), while similar association with humidity was reported in Australia, but with temperature having no effect on the virus transmission (7). The study from Brazil observed flattening of the temperature effect on the virus transmission at 25.8°C, thus suggesting that warmer weather will not cause the transmission decline, which is in accordance with the studies from Iran and Spain where they observed no changes in transmission rates under different temperatures and humidity (8, 9). These studies are inconsistent and do not give clear evidence as to whether there is an association between the temperature, humidity, and virus transmission, and the global view seems to give a clearer conclusion; all the three studies that conducted analysis at the global level found an association between higher humidity, warmer temperatures, and lower transmission rate (10). However, climate-dependent epidemic modeling suggested that the absence of population immunity is a much stronger factor in viral transmission and that summer weather will not substantially limit the spread of the COVID-19 pandemic (11). This is consistent with high numbers of infected individuals in tropical countries and the increase of cases in the south of the United States in the second half of June 2020.

Recent studies report increasing numbers of SARS-Cov-2 positive asymptomatic individuals (1), but it is not clear whether the apparent increase in people with mild or no symptoms is due to the change in the extent of testing, or some other characteristic of the SARS-CoV-2 virus. Aiming to evaluate the association of humidity and ambient temperature with the severity of the COVID-19 disease, we analyzed individual-patient data for 6,914 patients with COVID-19 admitted to hospitals in Bergamo, Italy; Barcelona, Spain; Coburg, Germany; Helsinki, Finland; Milan, Italy; Nottingham, United Kingdom; Warsaw, Poland; Zagreb, Croatia; and Zhejiang province, China since the beginning of the pandemic and compared it to environmental temperature and calculated indoor humidity. Furthermore, we analyzed information about COVID-19 severity from the COVID Symptom Study application that is collecting information of 37,187 individuals in the UK.

## Methods

### Studied Cohorts

We collected information about hospital admission, discharge dates, admission to the intensive care unit (ICU), need for mechanical ventilation, and type of discharge (alive or dead) for 5,229 successive patients hospitalized for COVID-19 in six European Hospitals and 13 hospitals in Zhejiang province, China since the beginning of the pandemic (Table 1). We included patients with confirmed diagnosis of COVID-19 at the time of admission. We confirmed that patients had a positive result on polymerase chain reaction testing of a nasopharyngeal sample and/or a clinically/radiologically diagnosis of COVID-19. Patients were not followed after discharge, but COVID-19 related early readmissions were considered as part of the COVID-19 course. The study protocol conformed to the ethical guidelines of the 1975 Declaration of Helsinki. In Zhejiang hospitals, ASST Papa Giovanni XXIII Hospital in Bergamo, Hospital del Mar in Barcelona and Helsinki University Hospital local ethics committees approved this retrospective study of COVID-19 patient data. For REGIOMED Hospital in Coburg, the Ethics Committee of the Bavarian state physician's association approved the study. In Nottingham University Hospital's trust, ASST GOM Niguarda, Warsaw, and Zagreb, this information was released as public statistical information.

**Table 1:** Basic information about included patients.

**Cohort name**	**Hospital name**	**Total number of patients**	**Included in the study**	**Sex** **(f/m)**	**Age** **(median, range)**
Barcelona	Hospital del Mar	1,999	1,786	969/817	57 (17–101)
Bergamo	ASST Papa Giovanni XXIII Hospital	2,249	995	265/730	70 (6–95)
Coburg	REGIOMED	89	89	48/41	75 (18–98)
Helsinki		100	100	44/56	54.5 (16–84)
Milan	Asst GOM Niguarda	713	685	242/443	63 (0–96)
Nottingham	Nottingham University Hospitals	795	795	356/439	75 (0–102)
Warsaw	Central Clinical Hospital of Ministry of the Interior and Administration	122	122	45/77	69 (19–96)
Zagreb	Clinical Hospital Dubrava & University Hospital for Infectious Diseases	237	237	93/144	63 (22–99)
Zhejiang		610	608	297/311	49 (18–93)

### COVID Symptom Study Application

The COVID Symptom Study app (12) developed by Zoe with scientific input from researchers and clinicians at King's College London and Massachusetts General Hospital (https://covid.joinzoe.com/) was launched in the UK on March 24, 2020 and, in 3 months, reached more than 3.9 million subscribers. It enables capture of self-reported information related to COVID-19 infections, as reported previously (12). Importantly, participants enrolled in ongoing epidemiologic studies, clinical cohorts, or clinical trials can provide informed consent to link data collected through the app in a HIPAA and GDPR-compliant manner with extant study data they have previously provided or may provide in the future. The Ethics for the app has been approved by King's College London Ethics Committee (REMAS ID 18210, review reference LRS-19/20-18210) and all users provided consent for non-commercial use. For this work, we included participants from the United Kingdom who started reporting with a healthy status and subsequently developed symptoms leading to suspect COVID-19 following the disease score presented in Menni et al. (12). In order to get an estimate of disease duration, the time for disease end corresponded to either the last day of report before stopping using the app, or the first healthy day when followed by six consecutive days of healthy reporting. To avoid censoring, only participants with a disease duration of <30 days and with a disease onset occurring before May 17 were included in the analysis (37,187 individuals). Severity score was calculated as a weighted average of symptoms at disease peak using as weight the normalized ratio in symptom frequency at disease peak between people reporting hospital visit after disease onset and those that did not.

### Data Related to Seasonal Changes

Ambient temperature data were obtained from the Climate Data Online [National Centers for Environmental Information (NCEI) database]: https://www.ncdc.noaa.gov/cdo-web/.

### Statistical Methods

The data collated from seven cohorts are summarized in Table 1. Patients without information about outcome were excluded from the analysis. Logistic regression was used to estimate the effect of admission date and local ambient temperature on mortality change. The following patient characteristics and hospitalization episode co-variates were explored: Died/discharged outcome was used as dependent variable and admission as independent variable along with age (in years) and gender (female/male). We then used the same approach for estimating the effect of ambient temperature on need for admission to ICU and for mechanical ventilation therapy. A linear model was then used to estimate the effect of ambient temperature on the hospital stay length (in days) as dependent variable and admission date as independent variable along with age and gender. Prior to the analysis, data transformation was undertaken with hospital length of stay increased by 1 (due to zeros) and log_10_ transformed (zero days in hospital stay correspond to hospitalization with a length lower than 24 h). Linear regression using median duration as dependent variable and 2-week period as independent variable was fitted to assess change over time.

For each dependent variable, raw data were presented with bar plots (death, ICU, and mechanical ventilation) or box-and-whiskers plots (hospital length of stay) for patients in 2-week groups. Fill of bars and boxes reflects the number of patients admitted to the hospital in a particular 2-week group. With groups of less than five patients, individual data points were plotted.

Coefficients estimated in logistic regressions and linear regression were combined using an inverse variance-weighted meta-analysis methods where, given the heterogeneity of cohorts, random effects models were used (R package “metaphor”).

Results of the meta-analysis were presented as forest plots, created using R package “ggplot2.” All statistical analyses were performed in R programming software (version 3.6.3), with the exception of logistic and linear regressions on Milano cohort data, which are performed in Stata Statistical Software (version 12) and the COVID Symptom Study cohort for which linear regression was performed using python statsmodels package (version 0.11.1).

## Results

**Figure 1:**
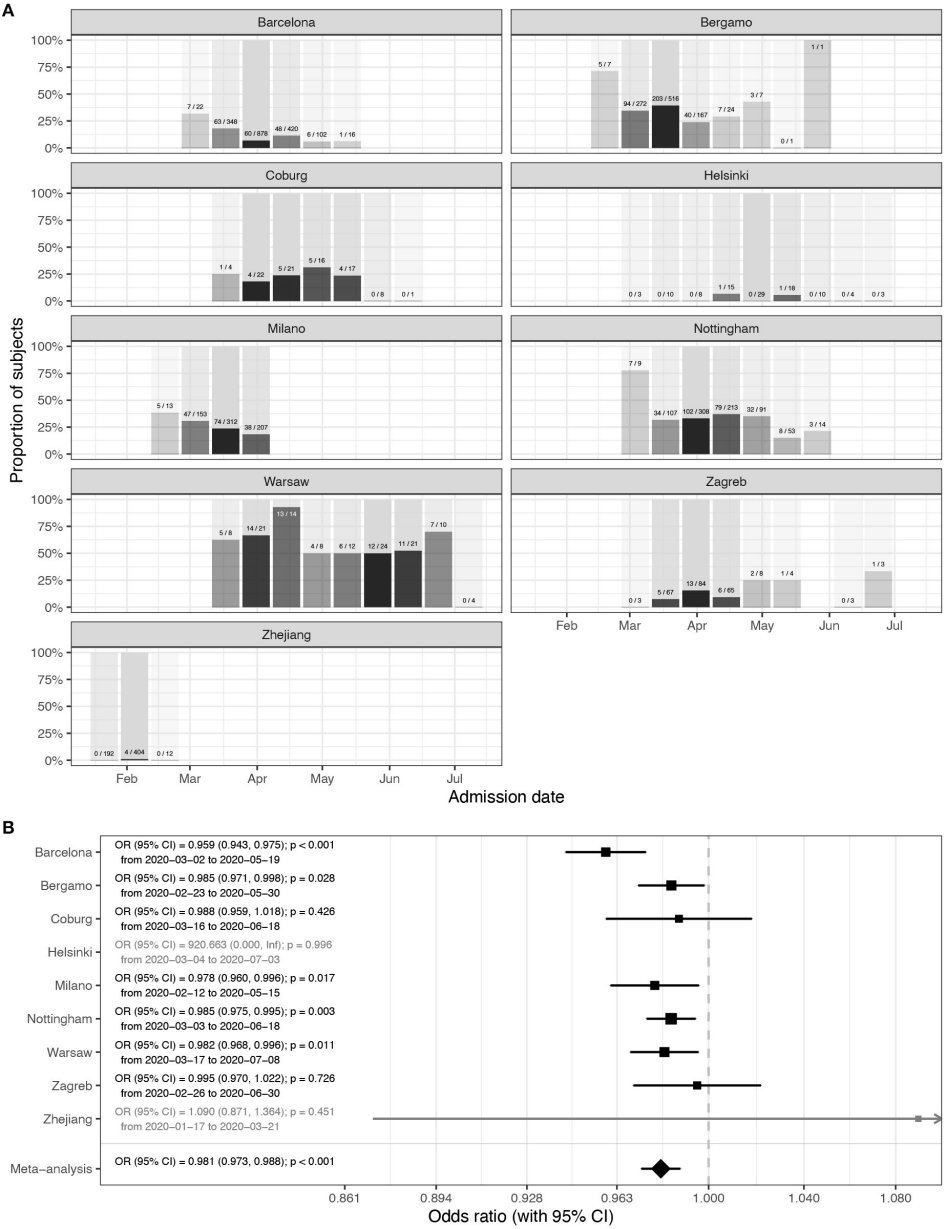
Mortality in people admitted in hospitals with COVID-19. **(A)** Hospitalization outcome (death/discharge) depending on the admission date (grouped in 2-week intervals) since the beginning of the pandemic. **(B)** Meta-analysis of the effects of admission date on mortality (presented as odds ratios per 1-day increase in admission date). In Helsinki, there were only two deaths, and in Zhejiang hospitals, four deaths, so they were not included in the meta-analysis. OR, odds ratio; CI, confidence interval.

Aiming to evaluate the seasonal nature of COVID-19, we evaluated disease course in 6,914 individuals from nine cohorts admitted to hospitals in Europe and China (Table 1). To avoid sampling bias, all hospitalizations that resulted in either death or medical discharge were included in the analysis. Actual numbers of patients who died and patients who recovered (grouped in 2-week intervals) since the beginning of the epidemic until the final follow-up date for reliable data capture reporting final outcome was available are presented in Figure 1A for each of the hospitals. Meta-analysis of the effect of admission date on the mortality is presented in Figure 1B. The most significant change was observed in Barcelona, where mortality odds decreased by 4.1% per day (*p* < 0.001). Weighted average decrease in mortality odds across all studied hospitals was 1.9% per day (*p* < 0.001). Our model included age as a co-variate, so this change is unlikely to be accounted for by change in age of patients. To further confirm that age was not underlying the observed changes, we analyzed age of patients admitted to hospitals in different periods and demonstrated that change in the age of patients was not a factor that could explain the observed decrease in mortality (Supplementary Figure 1).

Since there is no standard measure or classification of COVID-19 severity used across all hospitals, to further evaluate disease severity, we analyzed secondary outcomes. We compared the duration of hospitalization, need for ICU and separately mechanical ventilation. Strong and statistically significant decrease in the duration of hospitalization was observed in Barcelona, Coburg, Milano, Nottingham, and Zagreb. In Helsinki, Warsaw, and Zhejiang, the change was in the same direction but was not statistically significant. The only outlier was Bergamo, where the change was in the opposite direction, but the change was not statistically significant (Supplementary Figure 2). In meta-analysis, the decrease in lengths of hospitalization was statistically significant (10^b^ = 0.995; CI = 0.991–0.998; *p* = 0.007). The odds to need of intensive care decreased in all hospitals in Europe and was individually statistically significant in all hospitals beside Bergamo, Helsinki, and Zagreb (Supplementary Figure 3). Meta-analysis of European hospitals estimated that the odds to need the intensive care decreased by 2.2% per day of change in the admission date (OR = 0.978; CI = 0.962–0.993; *p* = 0.008) and the odds to need mechanical ventilation decreased 2.1% (OR = 0.979; CI = 0.964–0.994; *p* = 0.008) per day of change in the admission date (Supplementary Figure 4).

**Figure 2:**
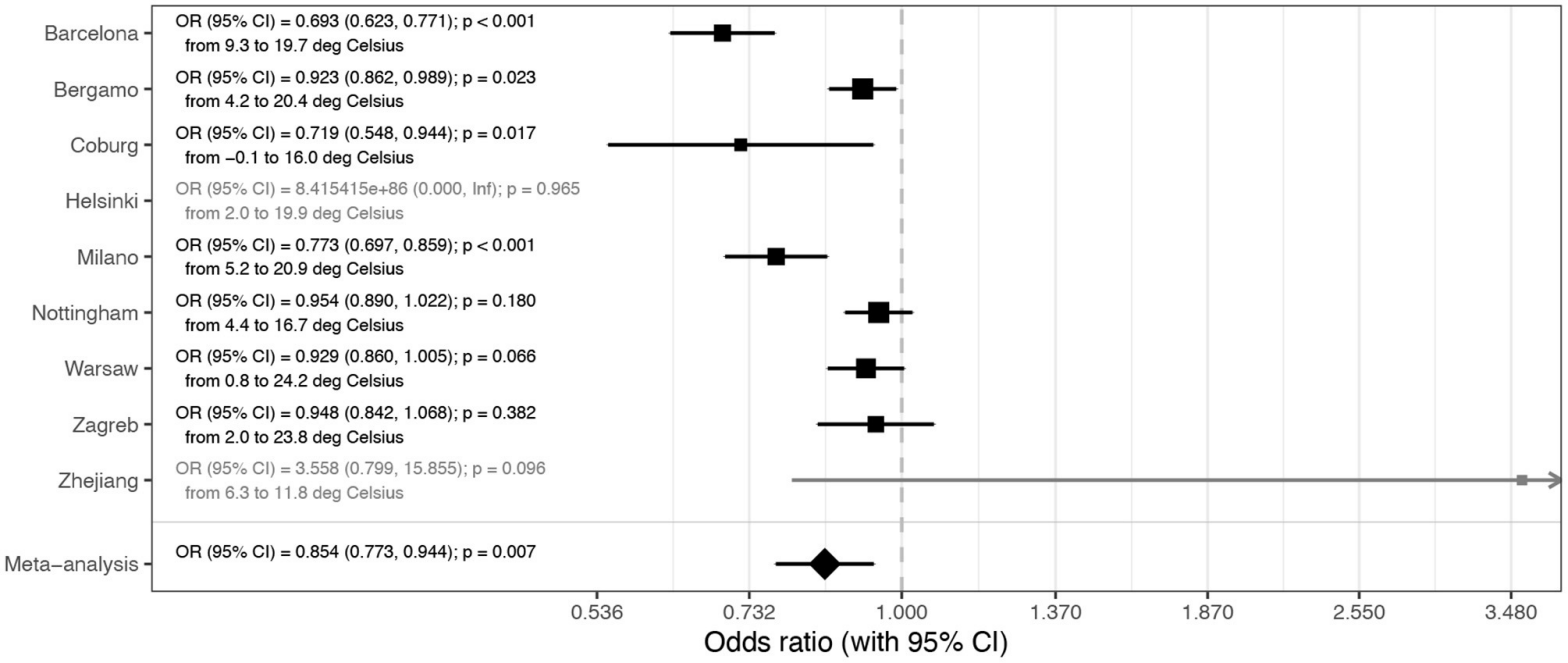
Meta-analysis of the effects of temperature on mortality (presented as odds ratios per 1°C increase in average daily temperature during hospitalization). In Helsinki, there were only two deaths, and in Zhejiang hospitals, four deaths, so they were not included in the meta-analysis. OR, odds ratio; CI, confidence interval.

While all hospitals in Europe were basically displaying the same trend of decreasing COVID-19 severity with time, in Zhejiang hospitals, there was either no change, or the changes trended non-significantly in opposite direction to European centers. The most notable difference between the COVID-19 pandemic in Europe and in China was that while the epidemic was entirely during winter in China, it covered both winter and spring periods in Europe. To evaluate whether weather was an important factor, we correlated the observed changes with local ambient temperature. Minimal and maximal local temperatures for all hospitals are presented in Supplementary Figure 5. To evaluate whether the change in temperature may have been responsible for the observed changes in disease severity, we modeled mortality with ambient temperature instead of admission date. The results presented in Figure 2 suggest a strong effect of ambient temperature on the mortality risk (OR = 0.854 per 1°C; CI = 0.773–0.944; *p* = 0.007).

**Figure 3:**
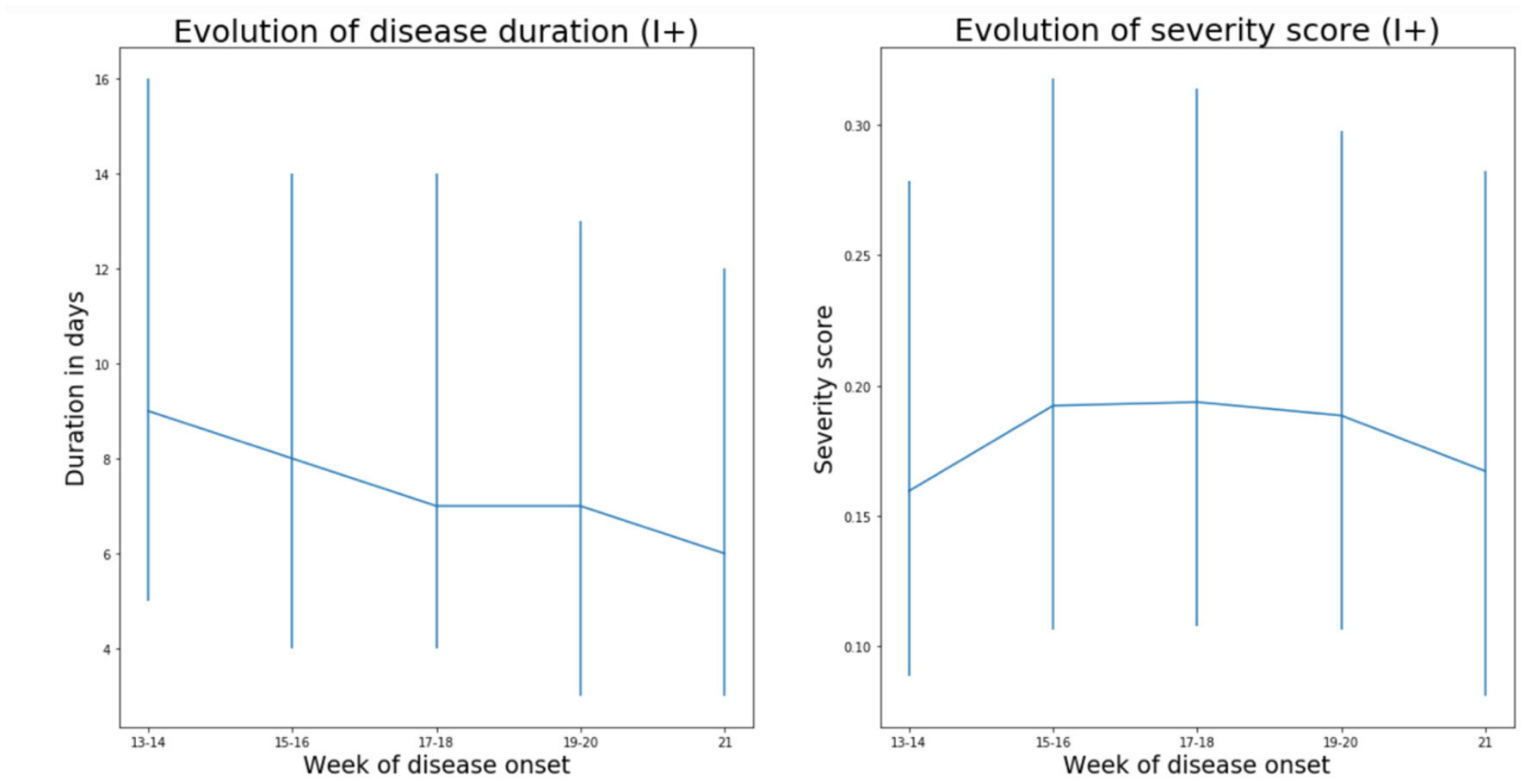
Data from 37,187 individuals/suspected COVID positives (I+) recording symptoms in the COVID Symptom Study application in the United Kingdom suggest that both severity (measured as weighted sum of symptoms accounting for difference at disease peak between those reporting hospital visit and those who do not) and duration of disease symptoms slightly decrease in the United Kingdom (median values and interquartile ranges are shown). Imputed status defined as per the application of the predictive model described by Menni et al. (12) was chosen over definite PCR diagnostic in order to avoid confounding factor of test access policy changes.

To further verify the change of COVID-19 with time, we analyzed individual symptom data for 37,187 participants of the COVID Symptom study app. Although there is also a sampling bias in that study, it is different from bias in hospitalization, so it was reassuring to observe a gradual decrease in duration of symptoms and COVID-19 severity in April and May (Figure 3). An assessment of the slope of duration as a function of time (2 ISO week) showed a significant decrease in duration (*B* = −0.7 *p* = 0.006). Regarding severity, while not overall significant (−0.0014 *p* = 0.836), the trend toward a decrease was stronger when considering the latest period (point 1 to the end slope = −0.0112 *p* = 0.116).

## Discussion

By analyzing hospital records of 6,914 patients admitted to eight European hospitals, we observed a strong and statistically significant decrease in COVID-19 mortality and severity with time. Possible change in the average age of patients in different stages of the pandemic is the first obvious explanation for the decreased severity, since age is the strongest predictor of COVID-19 severity [with up to 100-fold difference in mortality risk (13)]. However, age was included in our model as a co-variate and furthermore the average age of patients did not change with time (Supplementary Figure 2), so we excluded this hypothesis. An alternative explanation could be that there was change in policies for admission and/or release of COVID-19 patients during the evaluated period—possibly due to “overwhelming” medical facilities. This might have been particularly relevant in the situation of limited hospital capacity, when hospitalization may have been preceded with a triage process to identify patients who might benefit from hospitalization, admission to ICU or mechanical ventilation. However, the only hospital in our cohort that reached full capacity was Bergamo, while all others operated well below the maximal capacity for either hospitalization, or ICU, which suggests that changes in hospital admission policy were not a major driver behind the observed change in COVID-19 mortality and severity. This conclusion is further supported by concurrent decrease in duration and severity of symptoms of non-hospitalized individuals reporting symptoms in the COVID-Symptom Study Application (Figure 3). Change in COVID-19 management, also, could have resulted in decreased severity. However, all these changes were hospital-specific, and in the analyzed period, the most effective improvement in therapy was the introduction of dexamethasone, which was reported to reduce mortality from 24.6 to 21.6% (14). As we are learning more about COVID-19, patients are receiving better and better treatment, but the progress so far was not too large, which is particularly evident from the increased mortality in the second wave in Australia [case fatality rate (CFR) was 0.5% in the first wave (15) and 3.1% in the second wave (16)]. Therefore, it is hard to imagine that minor modifications in patient management could have significantly contributed to the observed decrease in disease mortality and severity in Europe.

After excluding these three causes for a Europe-wide decrease in disease severity and mortality in the period from March to June, the change in season surfaced as the most probable explanation since in all studied locations ambient temperature increased considerably in that period (Supplementary Figure 5). Exchanging hospital admission date with local temperature (Figure 2) showed that temperature strongly correlated with decrease in COVID-19 mortality. Since reverse causation is not possible, it is reasonable to conclude that COVID-19 as a disease has a strong seasonal nature. Despite the fact that most human coronaviruses are highly seasonal (17), the seasonal nature of COVID-19 is frequently challenged with the fact that numerous cases have been reported in tropical countries and that virus evidently can also be efficiently transmitted in hot and humid climates. However, in all these countries, the disease mortality and severity are very low (e.g., Singapore reported 26 deaths and over 44,000 confirmed infections), which actually suggests that there may be seasonal or climate-related differences in severity of COVID-19. It is possible that the same is the case for other respiratory viruses that show strong seasonality, but asymptomatic people are generally not tested for the presence of viral RNA in the nose; thus, viral transmission, outside of their season, was not observed. The notable exception that confirms this hypothesis was the 2009 swine flu pandemic in England when numerous PCR tests were also performed in the summer. These tests revealed infection in over 250,000 people in the summer wave, but with much lower mortality than in the wither wave (18). The large increase in the number of people with positive SARS-CoV-2 PCR tests in Europe in late summer and early autumn 2020 is not accompanied by the corresponding increase in deaths. The increase in number of cases and change in the age distribution of patients (19) have been suggested as possible explanation for the missing deaths. However, in Australia, which has reverse seasons, the situation was the opposite, and the mortality was much higher in the second (winter) wave of the pandemic. Despite increased testing and global increase in the knowledge on how to treat patients, CFR in the second (winter) wave was six times higher than in the first (summer) wave [“winter” CFR was 3.1% (16) compared to “summer” CFR, which was only 0.5% (15)].

It is very difficult to prove causality in an observational study, particularly when many correlated factors are changed in the same time, but the observed decrease in COVID-19 severity with the end of winter fits very well with the known effects of outside temperature on indoor humidity and consequential restoration of mucosal barrier function, which is often impaired by dry air during the heating season (20). Most respiratory viruses peak in winter and fluctuation of temperature and humidity has been proposed as the most potent drivers of seasonality, especially in the context of the epidemic in the winter season (17). However, the peak of infection and the severity of the disease are not always fully aligned. For example, although infection rates of rhinoviruses peak in spring and fall, the disease severity increases in winter (21). Seasonal appearance of respiratory viruses is often attributed to seasonal indoor crowding and effects of temperature and humidity on stability of viral particles (22), with effect of low air humidity on the mucosal barrier often neglected.

While often considered to be a physical barrier, mucus is actually an active biological barrier that crosslinks viruses and bacteria to mucins, a group of highly glycosylated proteins that are secreted to our mucosal barriers where they self-assemble into long polymers (23). Mucin glycans mimic cell surface glycosylation and, by acting as a decoy for viral lectins, trap viral particles, which are then transported out of airways by mucociliary clearance (24). Furthermore, since all envelope viruses are highly glycosylated, a number of lectins like trefoil factors (TFF) are secreted to mucous where they crosslink viruses by binding to glycans on both viruses and mucins (25). However, this barrier is functional only if it is well hydrated to both maintain its structural integrity and enable constant flow of mucus that remove viruses and other pathogens from our airways (24). If exposed to dry air, these barriers dry out and cannot perform their protective functions (26).

Animal experiments demonstrated the importance of humidity for both transfection of respiratory viruses and disease severity (27–29), while population-level studies in the United States indicated the importance of humidity for influenza transmission (30). One of these studies demonstrated that increasing relative humidity from 20 to 50% can significantly decrease mortality from influenza infections (29). In another study, humidification of air in obstructive sleep apnea patients reduced nasal symptoms by 60% (31), which all suggest that protective effects of humidity on mucosal barrier may be a dominant molecular mechanism behind seasonality of respiratory viruses.

A large part of human inter-individual differences are glycan-based and glycan diversity represent one of the main defenses of all higher organisms against pathogens (32). Glycans (which are covalently attached to most proteins) are chemical structures that are being inherited as complex traits, which enables diversity and significant inter-individual differences (33). SARS-CoV-2 spike glycoprotein is heavily glycosylated (34), and it was reported to bind to glycosaminoglycans (35) and sialylated glycans (36). ABO blood antigens are also glycans and are probably the best known example of glycan diversity; interestingly, people with blood type A and, thus, having one N-acetylgalactosamine more than type O are more susceptible to COVID-19 (37). All these suggest that, like most other viruses, SARS-CoV-2 is also dependent on glycans for transmission, which further support the importance of mucins and functional mucosal barrier in COVID-19.

Mucociliary dysfunction and respiratory barrier impairment promotes both initial infection and expansion of viruses within the airways of an infected individual (29). Dry air inhalation significantly decreases nasal mucociliary transition time (NMTT) in heathy individuals (38), affecting the duration of viral exposure on nasal mucosa. Nasal epithelial cells are the main portal for the initial infection and transmission of SARS-CoV-2 (39). Patients who developed clinically relevant infection after experimental transnasal viral challenge (Rhinovirus or Infl. B) had reduced epithelial barrier function (increased transepithelial resistance, reduced number of ciliated cells, and increased NMTT compared to those who were not infected or had a mild form) (40). However, experimental viral infection *in vitro* resulted only in decreased number of ciliated cells, without affecting tight junction protein expression (41). This controversy between *in vivo* and *in vitro* experiments suggests the importance of immune response in the control of epithelial barrier function (42). Recent studies on the interaction between climate changes and respiratory barrier dysfunction indicated not only higher incidence of viral infection but also higher vulnerability of nasal mucosa through increased incidence of nosebleed in the emergency departments in the conditions of low temperature and low humidity (43). A recently published study that exposed volunteers to respiratory syncytial virus (RSV), one of the pathogens responsible for the common cold, demonstrated that pre-existing inflammation in the respiratory mucosa was a risk factor for infection (44), which further supports the importance of mucociliary dysfunction and respiratory barrier impairment for infection with respiratory viruses.

## Limitations

Potential sampling bias is the main limitation of this study. By focusing on individual progression of the disease in already hospitalized patients, we excluded effects of the unknown number of true infections on national mortality rates, and we still cannot exclude the possibility that some other unidentified external factors (including confinement and social distancing, improvement and compliance of prevention and environmental hygiene protocols, and even decreased air pollution, which could have progressively affected the severity of patients arriving to the hospital) were affecting composition of hospitalized patient cohorts and contributing to the decreased COVID-19 severity and mortality. Therefore, it is important that tracking of individual symptoms in 37,187 UK patients are showing the same trend, since these are individuals voluntarily reporting symptoms and potential sampling bias that is independent from bias in hospitalization. The choice to include imputed positives was mostly motivated by the restriction in testing access that were observed over the first wave before being relaxed in May and June. Accounting only for PCR-tested positive reporting to the app would have unduly biased the results toward higher severity in the early days. We adopted instead the model developed by Menni et al. (12) that achieved a reasonable performance in prediction of positive cases (ROC-AUC 76%).

## Conclusions

Our data suggest that, in addition to affecting viral transmission, environmental factors also play an important role in already infected patients. Severity of COVID-19 decreased with the onset of spring, which paints a grim picture for the incoming winter and suggests that both disease severity and mortality may increase significantly. Since many hospitals have very dry air in winter, providing humidified air to patients in early stages of the disease may be beneficial. Considering the evident detrimental effect of dry air on our mucosal barrier and its role as the first line of defense against infection (45), in the situation of the rapidly progressing COVID-19 pandemic, it would be essential to actively promote universal humidification of dry air in all public and private heated spaces as well as active nasal hygiene and hydration (46). Humidity should also be monitored in cooled buildings with limited access to outside air, since air-conditioning is also an effective dehumidification and can result in very dry air.

## Data Availability Statement

The raw data supporting the conclusions of this article will be made available by the authors, without undue reservation.

## Ethics Statement

The studies involving human participants were reviewed and approved by the study protocol conformed to the ethical guidelines of the 1975 Declaration of Helsinki. In Zhejiang hospitals, ASST Papa Giovanni XXIII Hospital in Bergamo, Hospital del Mar in Barcelona and Helsinki University Hospital local ethics committees approved this retrospective study of COVID-19 patient data. For REGIOMED Hospital in Coburg, Ethics committee of the Bavarian state physician's association approved the study. In Nottingham University Hospital's trust, ASST GOM Niguarda, Warsaw and Zagreb this information was released as public statistical information. Written informed consent for participation was not required for this study in accordance with the national legislation and the institutional requirements.

## Author Contributions

GL designed the study and wrote first draft of the paper. DK, FV, IU, and CMe analyzed the data. DB, JV-G, IG, CSu, LL, MPoss, SB, NU, AMal, OE, CG, CR, LK, JPe, LO, BO, HY, TC, GA, CSt, AK, MK, OV, AS, RW, WW, ZK, AZ, KZ, MPost, IL, RČ, AMarko, JB, AMarkl, BM, SO, AV, JH, XC, JPa, MA, DP, TS, and CB collected and processed clinical data. All authors contributed to the article and approved the submitted version.

## Conflict of Interest

The authors declare that this study received funding from Zoe Global Ltd. The funder was not involved in the study design, collection, analysis, interpretation of data, the writing of this article or the decision to submit it for publication.
