# Supplementary material for: Effects of Environmental Factors on Severity and Mortality of COVID-19
Source: Front Med (Lausanne). 2021 Jan 20;7:607786. doi: 10.3389/fmed.2020.607786 (PMC7855590; doi:10.3389/fmed.2020.607786)
Supplement: Supplementary file 1 [file Image_1.pdf]

**Effects of environmental factors on severity and mortality of COVID-19**

Supplementary figures

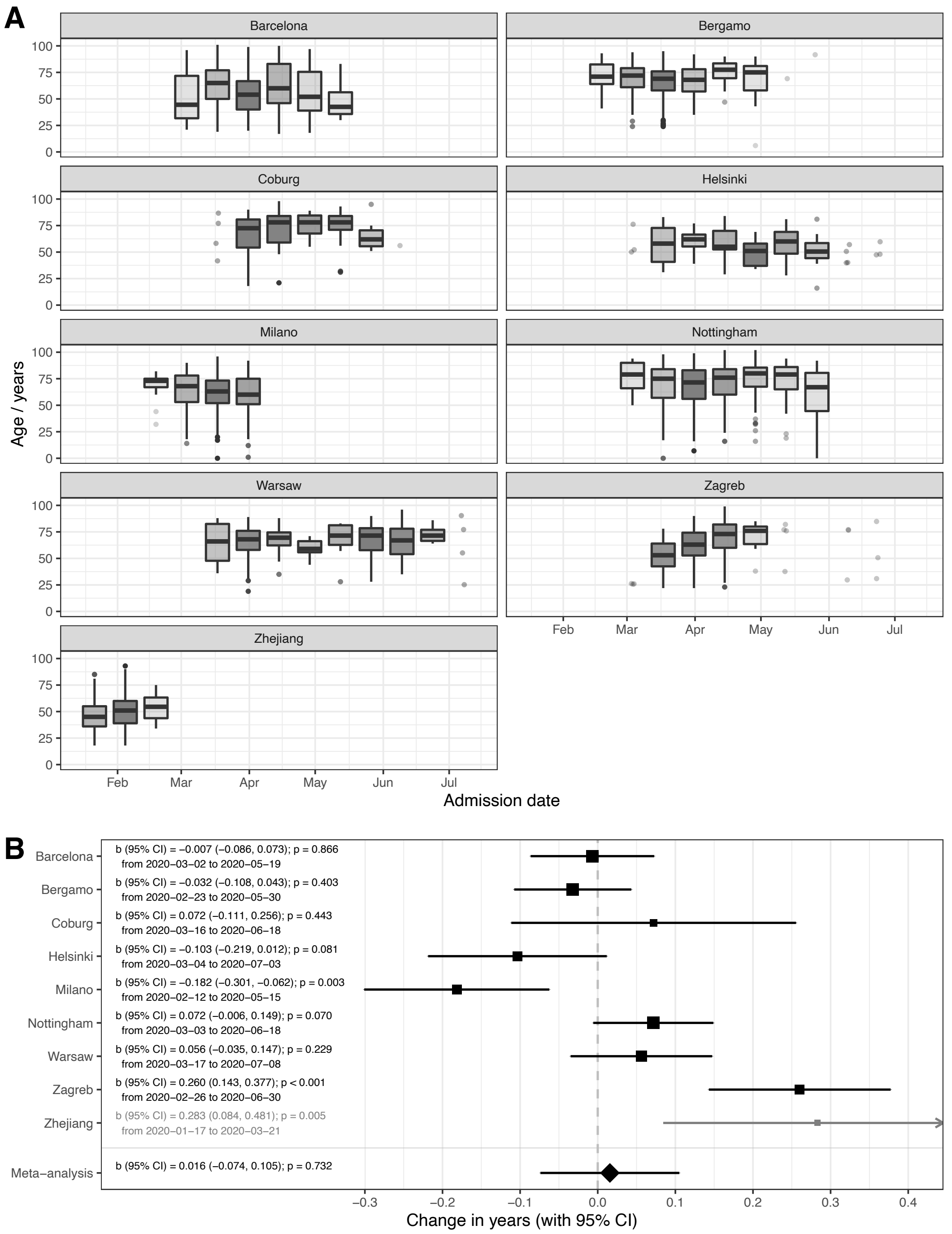


Supplementary Figure 1. Changes in age of admitted patients with time. A – age boxplots depending on the admission date (grouped in two-week intervals) since the beginning of the pandemics. Lower and upper limits of box present first and third quartile, respectively, and line within the box is median. Whisker lines extend to the minimum and maximum value within 'inner fence' defined as 1.5 times interquartile range bellow 1st and above 3rd quartile, respectively. Outliers are presented with dots. If two-week interval had 5 or less values data were presented with dots instead of boxplots; B - Meta-analysis of the effects of admission date on the mortality (presented as odds ratios per one day increase in admission date). In Helsinki there were only 2 deaths and in Zhejiang hospitals 4 deaths, so they were not included in the meta-analysis. OR – odds ratio, CI – confidence interval.

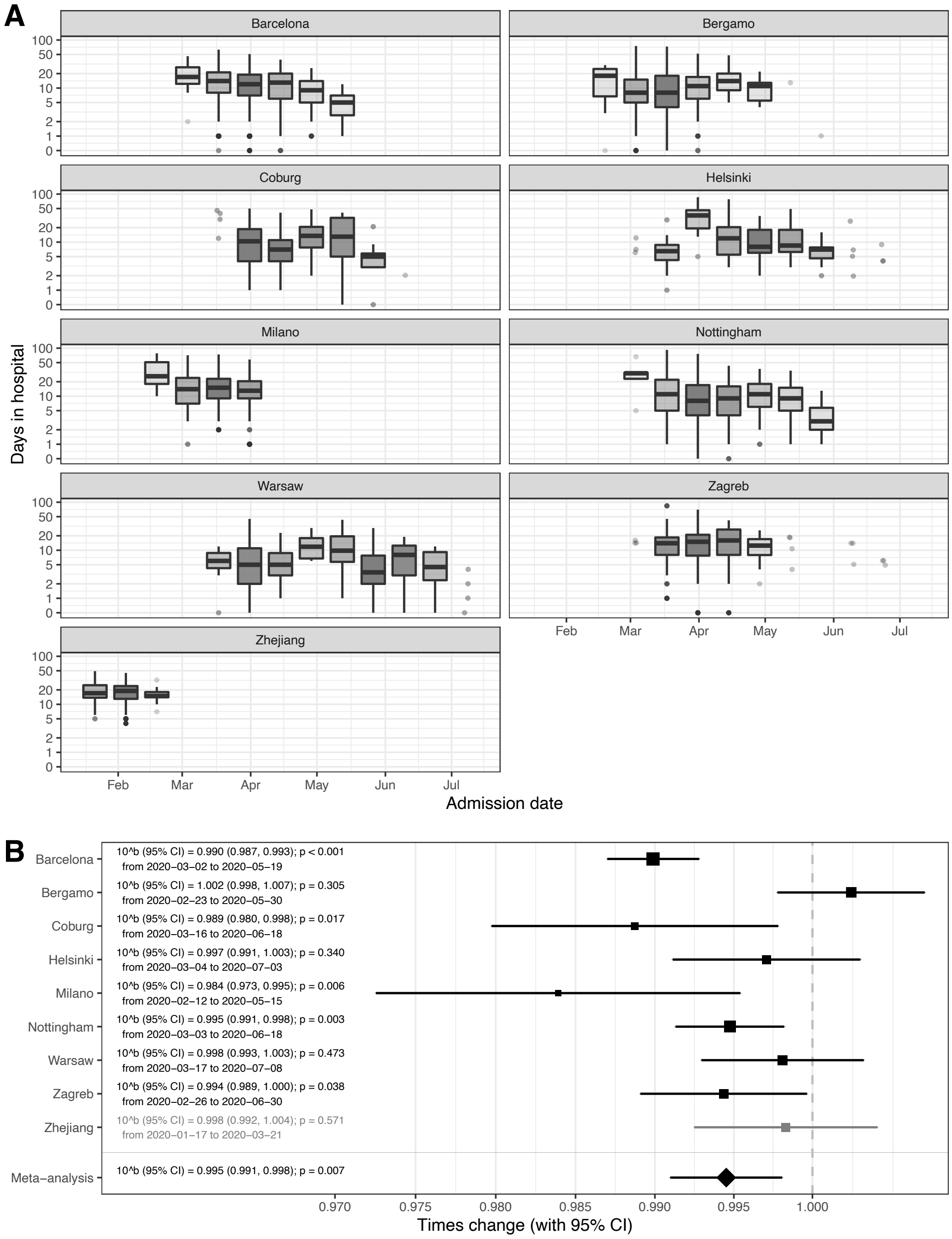


Supplementary Figure 2. Hospital stay of subjects admitted in hospitals with COVID-19. A - number of days stayed in hospital depending on the admission date (grouped in two-week intervals) since the beginning of the pandemics. Lower and upper limits of box present first and third quartile, respectively, and line within the box is median. Whisker lines extend to the minimum and maximum value within ‘inner fence’ defined as 1.5 times interquartile range bellow 1$^{st}$ and above 3$^{rd}$ quartile, respectively. Outliers are presented with dots. If two-week interval had 5 or less values data were presented with dots instead of boxplots; B - Meta-analysis of the effects of admission date on the hospital stay (presented as times change in duration per each one day increase in admission date). Zhejiang hospital in which all patients were admitted during winter was excluded from the meta-analysis. b – regression coefficient (back transformed), CI – confidence interval.

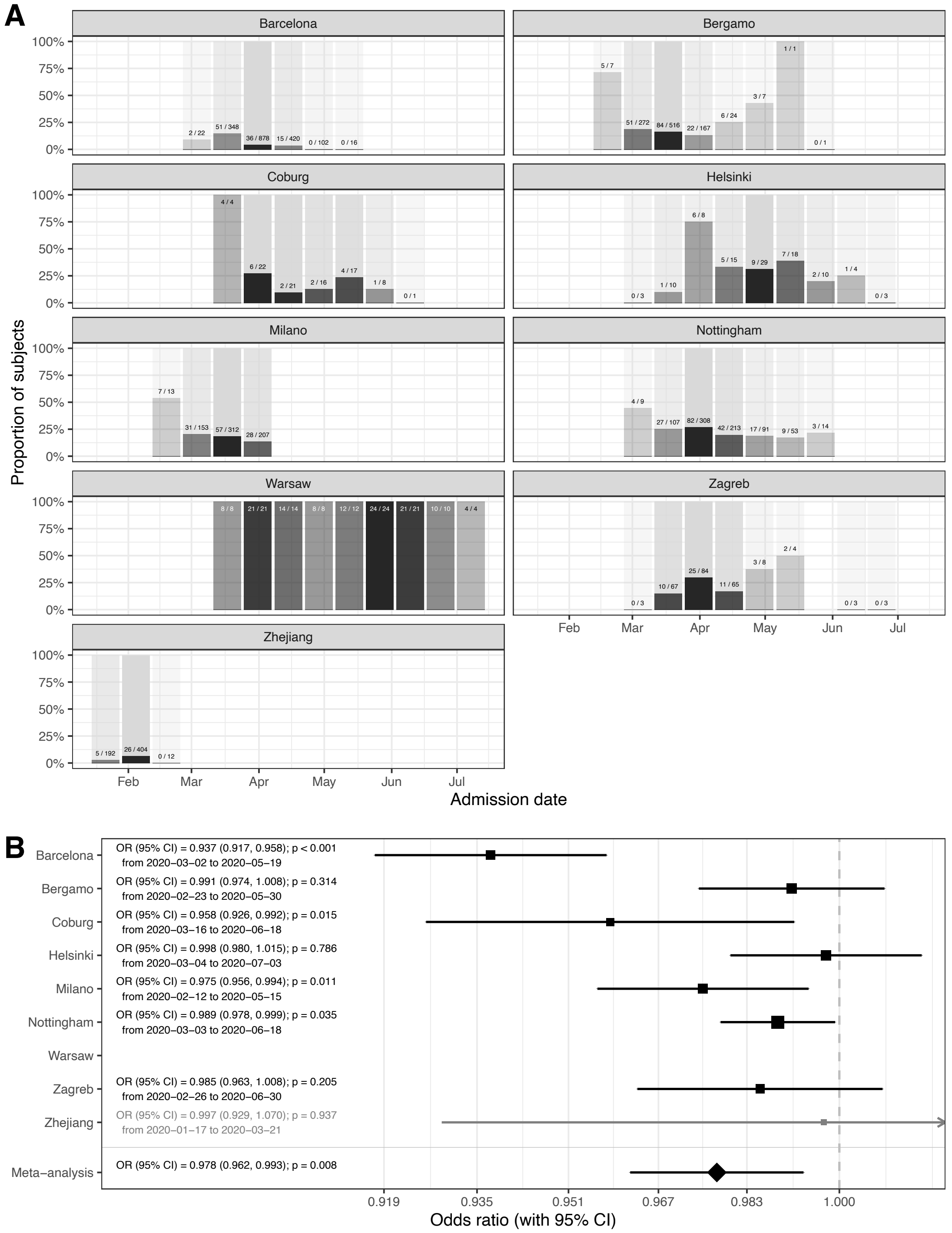


Supplementary Figure 3. Admission to the Intensive care unit (ICU risk) in people admitted in hospitals with COVID-19. A – proportion of people who were ever admitted to ICU depending on the admission date (grouped in two-week intervals) since the beginning of the pandemics; B - Meta-analysis of the effects of admission date on the ICU admission (presented as odds ratios per one day increase in admission date). Effect of the admission date was not calculated for Warsaw because all subjects were all in ICU. Time period of Zhejiang did not include both cold and warm weather and because of that results were not included in meta-analysis. OR – odds ratio, CI – confidence interval.

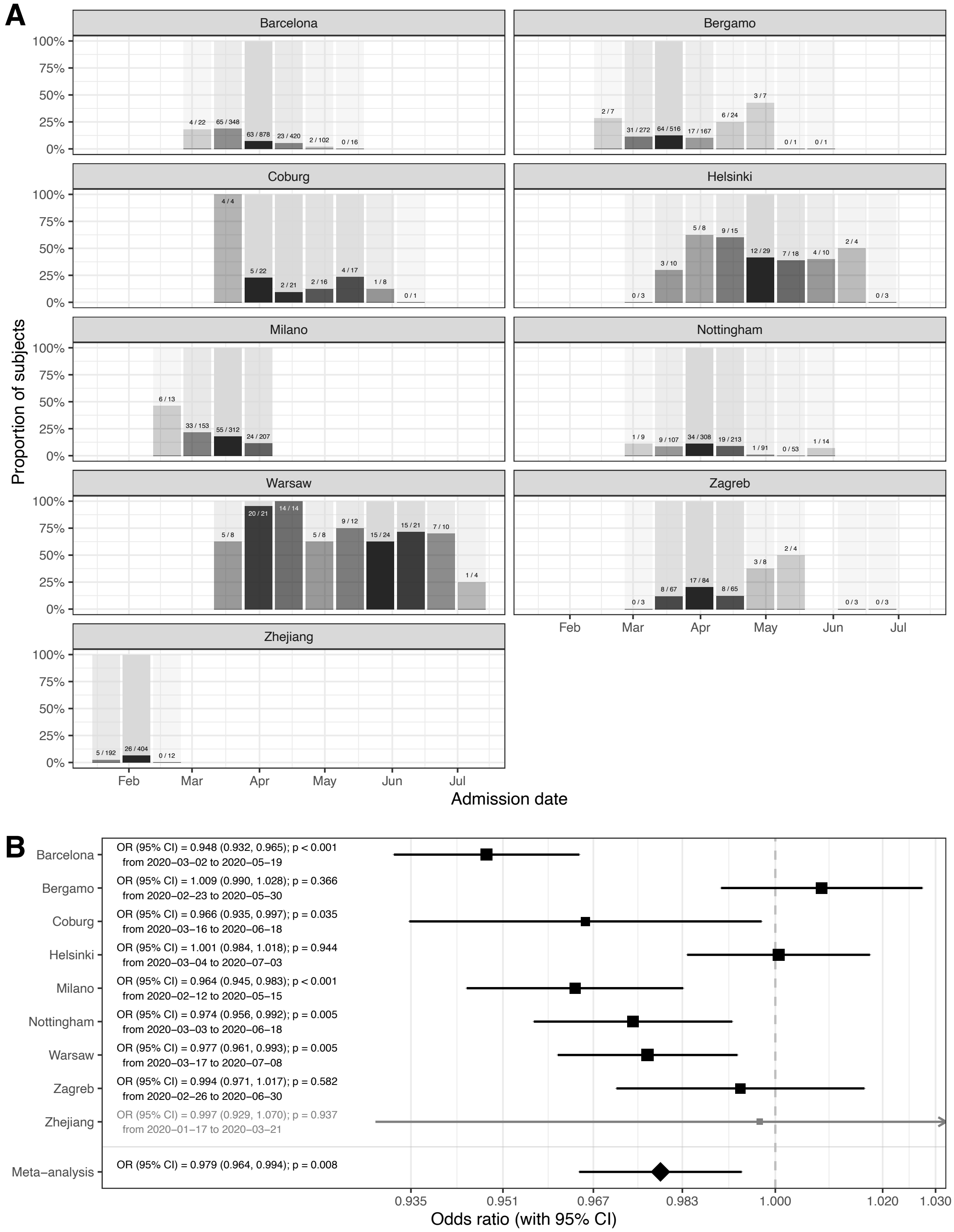


Supplementary Figure 4. Mechanical ventilation (ventilation risk) in people admitted in hospitals with COVID-19. A – Proportion of people who needed mechanical ventilation depending on the admission date (grouped in two-week intervals) since the beginning of the pandemics; B - Meta-analysis of the effects of admission date on need for mechanical ventilation (presented as odds ratios per one day increase in admission date). Time period of Zhejiang did not include both cold and warm weather and because of that results were not included in meta-analysis. OR – odds ratio, CI – confidence interval.

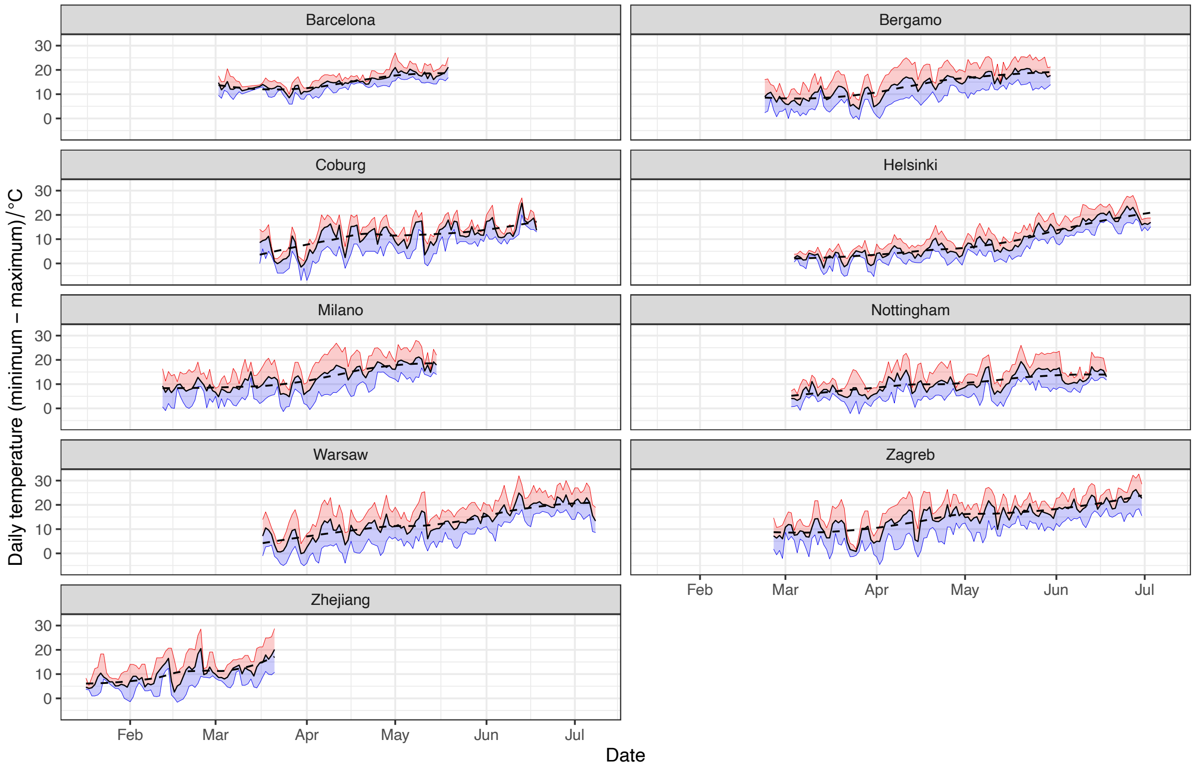


Supplementary Figure 5. Daily ambient temperatures in the study period. Solid black line – average daily temperature, red line – daily maximum, blue line – daily minimum. Dashed black line – locally estimated scatterplot smoothing of daily average temperature. Data were obtained from the Climate Data Online (National Centers for Environmental Information (NCEI) database): https://www.ncdc.noaa.gov/cdo-web/
